# Assessment of Physical Activity and Energy Expenditure: An Overview of Objective Measures

**DOI:** 10.3389/fnut.2014.00005

**Published:** 2014-06-16

**Authors:** Andrew P. Hills, Najat Mokhtar, Nuala M. Byrne

**Affiliations:** ^1^Centre for Nutrition and Exercise, Mater Research Institute, University of Queensland, South Brisbane, QLD, Australia; ^2^Griffith Health Institute, Griffith University, Gold Coast, QLD, Australia; ^3^Nutritional and Health-Related Environmental Studies Section, International Atomic Energy Agency, Vienna, Austria; ^4^Faculty of Health Sciences and Medicine, Bond University, Gold Coast, QLD, Australia

**Keywords:** physical activity assessment, human energy expenditure, objective measurement techniques, stable isotopes, accelerometry

## Abstract

The ability to assess energy expenditure (EE) and estimate physical activity (PA) in free-living individuals is extremely important in the global context of non-communicable diseases including malnutrition, overnutrition (obesity), and diabetes. It is also important to appreciate that PA and EE are different constructs with PA defined as any bodily movement that results in EE and accordingly, energy is expended as a result of PA. However, total energy expenditure, best assessed using the criterion doubly labeled water (DLW) technique, includes components in addition to physical activity energy expenditure, namely resting energy expenditure and the thermic effect of food. Given the large number of assessment techniques currently used to estimate PA in humans, it is imperative to understand the relative merits of each. The goal of this review is to provide information on the utility and limitations of a range of objective measures of PA and their relationship with EE. The measures discussed include those based on EE or oxygen uptake including DLW, activity energy expenditure, physical activity level, and metabolic equivalent; those based on heart rate monitoring and motion sensors; and because of their widespread use, selected subjective measures.

## Introduction

Physical activity measurement approaches are commonly used to quantify the amount and type of movement undertaken by individuals in different settings. In many cases, objective physical activity (PA) measurement approaches are also used to predict energy expenditure (EE). The ability to estimate PA and assess EE in free-living individuals is extremely important in the global context of increasing rates of obesity and type 2 diabetes mellitus and other non-communicable diseases (NCDs).

It is important to appreciate that PA and EE are different constructs. PA is defined as any bodily movement that results in EE (1) and accordingly, energy is expended as a result of PA. Simply stated, PA is a behavior that results in an elevation of EE above resting levels. The terms are often considered synonymous but are inherently different and can be assessed using different approaches (2). The criterion or “gold standard” approach to assess total energy expenditure (TEE) in a free-living context is the doubly labeled water (DLW) technique. TEE is comprised of multiple components including physical activity energy expenditure (PAEE), resting energy expenditure (REE), and the thermic effect of food (TEF). Despite PA being a complex and multifaceted construct measured using many approaches, unlike for the measurement of TEE, there is no recognized “gold standard” technique (3, 4).

A large number of objective measurement approaches are available to quantify PA and EE of different populations. However, the accurate measurement of EE and PA in many groups, including children, is very challenging (5, 6) due to their intermittent and often sporadic movement (7). Because PA is a complex and multidimensional behavior, precise quantification can be difficult (8). A major challenge in PA and nutritional epidemiology is the choice of the most accurate and objective measure suitable for large populations (8, 9). The choice of assessment approach for both PA and EE is influenced by numerous factors including affordability and participant burden (10). Additional factors include the age of participants, sample size, assessment time frame, the type of PA information required, data management options, and measurement error associated with the approach (11–14). Fundamentally, all measurement techniques have inherent strengths and limitations, and there is often value in using combined approaches. Some of the important considerations include the following:

- How was the technique derived – was it based on EE, heart rate (HR), or accelerometry data?
- What is the cost of the technique and how practical or convenient is it for participants and investigators?
- What is the intended use, for example to assess the impact of PA on energy balance, metabolic health or a component of fitness, and in a small or large-scale study?

The goal of this review is to provide information on the utility and limitations of a range of objective measures of PA and their relationship with EE. Definitions of major terminology are followed by an overview of selected objective approaches to assess PA and EE. Measures discussed include those based on EE or oxygen uptake including DLW, activity energy expenditure (AEE), physical activity level (PAL), and metabolic equivalent (MET); those based on HR monitoring and motion sensors (pedometers and accelerometers); and because of their widespread use, subjective measures including selected physical activity questionnaires (PAQs).

## Terminology

Inconsistent use of terminology has impacted the fields of nutrition, PA, exercise, and EE assessment, therefore, an overview of key terminology is a useful starting point. PA is a global term traditionally defined as bodily movement resulting from contraction of skeletal muscle that results in an increase in EE above resting levels (1). In turn, PA can be categorized on the basis of context or setting to include leisure-time or recreational PA and subcomponents of sport, transportation, and occupational activity. Alternatively, *exercise* is commonly defined as planned, structured, and repetitive movement with the intention of promoting or maintaining one or more components of physical fitness (1). PA and exercise can be quantified according to *intensity* (how hard?), *duration* (how long?), *frequency* (how often?), and *mode* (or type), such as walking, running, swimming, etc. (15). Howley (16) provides a useful overview of the various terms associated with PA and exercise plus guidelines for consistent interpretation of exercise intensity and volume.

As mentioned above, an outcome of participation in PA or exercise is the expenditure of energy, commonly quantified in terms of intensity. Intensity can be referenced in different ways (16). For example, intensity of aerobic exercise is typically referenced using increase in HR in beats per minute or the energy expended over and above the body’s resting requirements to quantify PA as AEE or as *exercise energy expenditure* (ExEE). Similarly, AEE and ExEE can be expressed relative to resting values where 1 MET at rest equates to 3.5 mL/O_2_/kg/min^−1^ or 1 kcal/kg/h. It is important to note that the conversion of MET to kilocalories can be erroneous when using these standard conversion factors (17–20). However, the use of a correction factor based on measured or predicted *resting metabolic rate* (RMR) can reduce this error in some activities (18). Further detail regarding the MET concept is provided in a later section.

**Figure 1:**
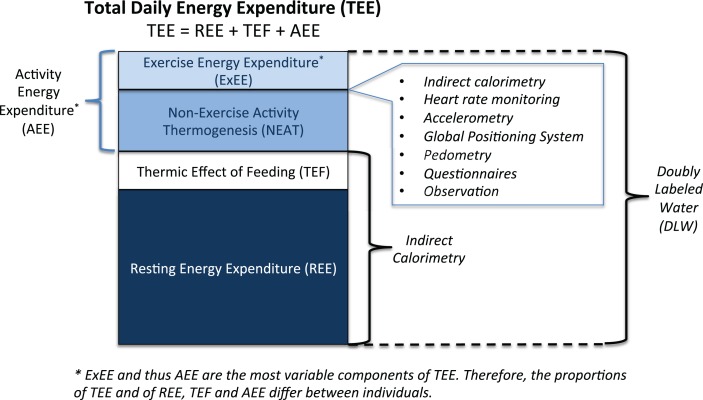
**Components of total daily energy expenditure and measurement approaches**.

Gross EE is quantified on the basis of oxygen consumption and is referenced in kilocalories per minute or kilojoules per minute. To be more appropriate to the individual, oxygen consumption should be expressed relative to body weight. Figure 1 portrays the subcomponents of TEE (discussed in more detail below), and also identifies the commonly used objective assessment techniques to quantify each subcomponent. Each technique will be discussed in detail in a later section.

## Components of Total Energy Expenditure

*Total daily energy expenditure* (TEE or TDEE) is comprised of REE, TEF also referred to as *diet-induced thermogenesis* (DIT), plus AEE.

Energy expenditure can be estimated by measuring macronutrient or oxygen consumption, or heat production or carbon dioxide production. Most measurement approaches in use today involve the measurement of oxygen consumption and/or production of carbon dioxide via *indirect calorimetry*. In contrast, *direct calorimetry*, the measurement of heat production in a metabolic chamber, is not widely utilized.

### Resting energy expenditure

Resting energy expenditure or RMR represents the largest proportion of TEE. Simply defined, REE represents the energy expended at rest by a fasted individual in a thermo-neutral environment. RMR is typically slightly higher than *basal metabolic rate* (BMR) that is measured under stricter conditions.

Estimation of energy requirements of individuals is typically undertaken by measuring RMR or REE or estimated using standard equations then multiplied by a known factor to derive an estimate of PAL.

### Determinants of REE

Major factors contributing to individual variation in REE include age, gender, body size, body composition, ethnicity, physical fitness level, hormonal status, and a range of genetic and environmental influences (21–25). Following is a brief overview of the first four determinants.

### Age

Typically, RMR reduces with age as a function of biological changes (26–28) including loss of lean body mass and associated metabolic activity (29).

### Gender

The bulk of the gender differences in REE are explained by differences in body composition with the typical adult female having more fat in proportion to muscle than males (21). Females have a metabolic rate 5–10% lower than males of the same height and weight. Reductions in REE occur later in women, at ~50 years of age compared to 40 years in men (27), and values are lower in women even after adjusting for differences in body composition (30, 31).

### Body size

Bigger individuals have more tissue and hence, higher energy requirements (greater metabolic activity) than smaller individuals. In addition to age, gender, body size, and body composition (proportion of fat and muscle), a range of factors can also contribute to inter-individual differences in metabolic rate including genetics, growth and repair of body tissues, ethnicity, and the environment.

### Body composition

Body composition plays a significant role in REE with the primary determinant being fat-free mass (FFM) (24). Therefore, most of the inter-individual variability in REE can be accounted for by differences in FFM (22). Along with age and gender, physical fitness has a major influence on the amount and proportion of FFM.

## Calorimetry

Human energy metabolism involves the production of energy from the combustion of fuel in the form of carbohydrate, protein, fat, or alcohol. In this process, oxygen is consumed and carbon dioxide is produced. The measurement of EE involves the measurement of heat production or heat loss directly, referred to as direct calorimetry. The measurement of a proxy of heat production or loss by measuring oxygen consumption and/or carbon dioxide production is called indirect calorimetry (32). Early calorimeters for the measurement of human EE were direct calorimeters; however, most measurement of EE today is via indirect calorimetry.

A relatively small number of metabolic (or respiratory) chambers are in use globally, however, there has been a resurgence in interest and increase in numbers in recent years (33). Such chambers are used to assess EE energy over extended periods, from 24-h to a number of days, and provide accurate measures of 24-h and sleeping EE plus long-term substrate utilization. However, because of the confined space, chambers do not provide an accurate estimate of an individual’s free-living AEE.

Metabolic chambers and small metabolic carts with ventilated hood systems are similar in their requirement of the measurement of CO_2_ and O_2_ concentrations and flow rate to calculate oxygen consumption, carbon dioxide production, *respiratory exchange ratio* (RER), and metabolic rate (34–36). Both systems measure oxygen consumption and carbon dioxide production continuously and this enables the accurate determination of energy production under controlled laboratory conditions.

Measurements with a ventilated hood are typically performed over a minimum of 30-min to several hours to determine REE or TEF. Measurements using a metabolic chamber typically last from a number of hours to several days and allow the determination of REE, TEF, and AEE for (standardized) PA. Typical ventilated hood systems use a transparent plastic canopy that encloses the head. Room air is drawn through the hood and the flow and concentration of oxygen and carbon dioxide in the intake and expired air are accurately measured and REE calculated (37). In summary, this approach is a form of indirect calorimetry as heat is not directly measured. Rather, the approach measures O_2_ consumption and CO_2_ production, which are then used to calculate EE.

Whole-room metabolic chambers or calorimeters provide accurate measurement of REE but as mentioned above, are less accurate in the assessment of the energy cost of PA undertaken in the chamber, typically due to physical constraints imposed by the size of the chamber. Most whole-body indirect calorimeters are ~15 m^3^ in volume and contain basic furniture such as a single bed, chair, table, television, exercise bicycle, sink, and toilet. EE calculations from O_2_ and CO_2_ exchanges typically use the equations of Livesey and Elia (38). Very recently, Lam et al. (33) developed a set of equations to prescribe and adjust energy intake to achieve energy balance in respiratory chambers over 24-h.

Methods of indirect calorimetry are the most commonly used to quantify human EE in both laboratory and field settings, typically by measuring O_2_ consumption. This approach is based on the relationship between O_2_ consumption and energy produced, i.e., for each liter of O_2_ consumed by the body, the equivalent of ~5 kcal is utilized. In simple terms, by measuring O_2_ consumption during defined tasks such as resting, standing, walking, and running, the energy cost or energy expended can be determined.

## Doubly Labeled Water Technique

The DLW technique is widely acknowledged as the criterion or “gold standard” approach to assess TEE (39–41). The technique is applicable in a wide range of populations including the most vulnerable such as pregnant and lactating women and infants. Importantly, the technique is suitable for use in a free-living context, is non-invasive and imposes minimal participant burden. TEE is typically assessed over a 7- to 14-day period (depending on the analysis approach and age of participant). Another major advantage is the accuracy and precision of the technique.

Despite being the criterion method for the assessment of TEE, the DLW technique does not provide specific information regarding daily PA (11). In short, the technique provides an accurate measure of TEE over a chosen number of days or weeks from which average daily EE can be calculated, but does not quantify activity type, intensity, or duration accounting for this EE (10). Similarly, as the analysis of biological samples (commonly urine) for the technique requires the use of sophisticated laboratory-based equipment, the combined cost of isotopes, and the analysis of samples is a potential impediment for large-scale studies. Despite these limitations, there is great scope for further use of this “gold standard” technique. Importantly, a further illustration of the utility of the technique is its use to validate other approaches for the quantification of free-living EE (14, 42). A recent paper confirmed the reproducibility of the DLW technique in longitudinal studies and validity of the technique to define energy intake plus monitor adherence and body composition changes across periods ranging from 2.5 to 4.4 years (43).

In the DLW technique, daily urine samples are collected over a 7- to 14-day period and subsequently analyzed using isotope ratio mass spectrometry (IRMS) (40). The stable isotopes, deuterium (^2^H) and oxygen-18 (^18^O) are administered orally via a drink of water, and elimination of the isotopes from the body is tracked (44–46). The difference between the elimination rates of ^2^H and ^18^O is equivalent to the rate of carbon dioxide production that can then be converted to average TDEE (47). Readers are encouraged to access a number of recent International Atomic Energy Agency (IAEA) publications for a detailed overview of the DLW technique for the assessment of TEE and other isotopic techniques in nutrition (40) and also recent papers in the area (44).

Components of TEE, including REE, are measured via indirect calorimetry (or using prediction equations) (48–50). This enables the subsequent calculation of AEE (assuming that TEF constitutes 10% of TEE):

$$\text{AEE }\left(\text{kcal}∕\text{day}\right)=0.\text{9}\times \text{TEE }\left(\text{kcal}∕\text{day}\right)-\text{REE }\left(\text{kcal}∕\text{day}\right)$$

Activity energy expenditure represents all energy expended above the resting level and energy costs associated with the ingestion and assimilation of food. AEE is a direct measure of the energy cost of PA and may be used to measure the energy cost of a specific task (as kilocalories per minute or kilojoules per minute) OR to estimate average EE (13):

$$\begin{array}{llll} \text{AEE }\left(\text{kcal}∕\text{day}\right) & =\text{total EE }\left(\text{TEE};\text{ kcal}∕\text{day}\right)\; & & \;\\ & \;-\text{Resting EE }\left(\text{REE};\text{ kcal}∕\text{day}\right)\; & & \; \end{array}$$

Activity energy expenditure is also influenced by body weight such that larger (heavier) individuals expend more energy at a given speed than smaller individuals. Exercise economy, or the efficiency of performing a movement task, also impacts EE (13).

As mentioned above, daily AEE can be calculated as an average across the DLW monitoring period, however, the technique does not provide any information regarding the mode, intensity, or duration of PA. Accordingly, we need to utilize a range of other objective measures to predict AEE and PAL.

## Physical Activity Level

Physical activity level is the ratio of TEE to REE (or BMR) and provides an index of the average relative excess output related to PA (intensity × duration) for a 24-h period (13) with TEE commonly derived using the DLW technique. The validity of PAL has been tested and confirmed in a large sample (51) and when derived from DLW data, the index is relatively accurate but expensive. Less expensive is the estimation of TEE using HR data, as discussed in a later section (13). Some have queried the relevance of a ratio between TEE and REE if PAL is not independent of body weight (52). In population studies, PAL is calculated by dividing total energy intake by an estimate of REE with total energy intake serving as a surrogate of TEE and assuming individuals are in energy balance. REE is measured or predicted from age, gender, height and weight (13).

Physical activity level can also be used to estimate total daily energy requirements of a population by assuming an average PAL for the group being studied. For example, a PAL equivalent to 1.56 would predict an average energy requirement of ~2000 kcal/day for women weighing 55 kg (53). The index has also been used to verify the accuracy of self-reported energy intake (54), that is, below a certain threshold (PAL × 1.2) the index suggests that energy intake is underestimated (13).

In well-nourished adults, the average PAL is a major determinant of energy requirements. PAL can be measured or estimated from average values of 24-h TEE and REE:

PAL=TEE∕REE

Typical PAL values in free-living adults range from 1.40 to ~2.40.

## Metabolic Equivalent

One MET equates with the oxygen consumption (O_2_) required at rest or sitting quietly and is assumed to be 3.5 mL/O_2_/min × kg body weight. The index is used to express O_2_ uptake or intensity of activities as multiples of the resting or 1 MET value and is useful for describing and prescribing exercise of different intensities (13, 55).

Comprehensive lists of EE estimations for numerous physical activities have been developed and published in a Compendium (17, 56, 57). This enables the estimation of daily EE by converting time spent in PA to energy equivalents. Activities range from 0.9 MET (sleeping) to 18 METs (running at 10.9 mph) (13). This classification system is particularly useful for epidemiological studies as MET scores can be ascribed to individuals according to their self-reported PA levels (58). However, the actual energy cost will vary between individuals due to differences in body mass, adiposity, age, gender, and environmental conditions (17). To allow for differences in body weight, the MET is generally expressed in terms of O_2_ uptake per unit body mass: 1 MET = ~3.5 mL/O_2_/min/kg × min (55).

As 5 kcal is ~1 L of oxygen consumed, 1 MET is equivalent to ~1.0 kcal/kg × h OR 4.184 kJ/kg × h.

The precision of this factorial method to quantify EE is influenced by two main factors. PA estimates are only as good as the information recorded, therefore, the accuracy of an individual’s recall of PA completed is a major influencing factor (55, 59–61). Secondly, EE estimates are influenced by the accuracy of the assigned MET level and the underlying premise of the factorial system, that is, the consistency of the assumed resting value of 3.5 mL/O_2_/min × kg body weight for individuals of different size and shape.

It is important to remember that the Compendium was developed to classify PA and standardize MET intensities in population health research, not to determine the precise energy cost of PA (17, 19, 56, 57). The MET system is widely used by researchers, clinicians, and practitioners; however, increasing evidence suggests that estimates of AEE using the factorial system may be inaccurate across individuals of different body mass and body fat category. In a heterogeneous sample of 769 weight-stable and healthy adults (18–74 years of age, 35–186 kg), the 1 MET value of 3.5 mL/kg × min overestimated the actual resting VO_2_ value by an average of 35% and the 1 MET of 1 kcal/kg × h overestimated REE by 20% (18). Very recently, Wilms et al. (62) confirmed that the commonly used 1 MET value largely overestimated EE in overweight to obese individuals and produced BMI-specific MET correction factors.

In summary, the most common utilization of the MET has been to categorize PA intensity. Moderate PA is defined as 3–6 METs, moderate-to-vigorous PA as >3 METs, and vigorous PA as >6 METs (3).

## Heart Rate Monitoring

Estimation of EE and PA via HR monitoring is popular, convenient, relatively inexpensive, non-invasive, and versatile. Along with pedometers and accelerometers, HR monitors are major examples of objective measurement (63). Monitoring HR minute-by-minute enables detailed information on frequency, intensity, and duration of free-living PA (13).

### Measuring energy expenditure

Heart rate monitoring is used to estimate EE based on the assumption of a linear relationship between HR and oxygen consumption (VO_2_). Despite considerable inter-individual variability in the slope of the HR–VO_2_ relationship, the linear relationship is consistent for an individual across a range of sub-maximal tasks (64, 65). Inter-individual differences are predominantly a reflection of differences in movement efficiency, age, and fitness.

The relationship between HR and EE for an individual is established using a sub-maximal calibration procedure typically undertaken immediately following the assessment of REE. HR and breath-by-breath VO_2_ and VCO_2_ are measured (averaged over 10-s intervals) using a metabolic cart in the following sequential steps: 5-min sitting, 5-min standing, 5-min cycling at low resistance (55 W), and further 5-min blocks of increasing cycling resistance while maintaining a cadence of 60 rpm. Cycling resistance is increased by 50 W in each subsequent 5-min block until the participant has cycled for at least three incremental stages, depending on level of fitness, and HR has reached ~150 beats/min. Average EE for each activity and at each workload is estimated from VO_2_ and VCO_2_ values using the equations of Livesey and Elia (38). To equate HR to EE, a regression line of HR to EE is developed for each individual from the sub-maximal calibration procedure and using measurements for sitting, standing, and at each of the workloads. The critical HR, below which the relationship between HR and EE is non-linear (flex-HR), is calculated from the mean of the highest HR when the participant was standing, and the lowest HR when exercising (66).

Consequently, once an individual or group regression line has been calculated, HR can be used to estimate oxygen consumption and EE in free-living conditions. A wide range of HR monitors, varying in sophistication and function, are now used in different contexts (67). Many HR monitors have significant storage capacity and the ability to record average HR data in 5-s or 1-min blocks across a week (68). A major advantage of using HR monitoring at the individual level is the ability to calibrate the monitor to the individual. Individualized HR–VO_2_ regression equations provide greater accuracy as they account for individual differences in health and fitness.

However, the method has important limitations. Because the relationship between HR–VO_2_ differs between upper-body and lower-body activities (69), the use of a single regression line derived from an activity such as walking or running will not be accurate for other activities. Further, while there is a very close relationship between HR and EE during exercise, this is not the case during rest and light activity (11, 66, 70). This problem can be overcome using the flex-HR method that utilizes an individually predetermined HR to discriminate between resting and exercise HR (71). HR monitoring has been validated for measurement of EE in controlled settings (72, 73) and free-living contexts (74, 75) in young people (5).

### Monitoring exercise intensity

A major advantage of HR data is the ability to quantify the intensity of exercise and estimate EE in continuous or steady state aerobic exercise. HR levels to describe exercise intensity should be expressed as %HR_Reserve_ and/or %HR_max_ enabling exercise intensity to be classified into six categories from very light to maximal (76). Using such an approach, it is possible to equate the HR category with its associated %VO_2max_ or %VO_2Reserve_ or MET and not require the measurement of VO_2_. However, it is much more accurate if the VO_2_–HR relationship is measured for each individual as outlined above (68).

In summary, HR is a major physiological marker for PA but is influenced by a wide range of factors unrelated to the activity being monitored. As such, HR provides an estimate or overview of PA but estimates can be improved if used in conjunction with other devices such as accelerometers (11).

## Motion Sensors

### Pedometers

Pedometers are arguably the most popular and widely utilized form of motion sensor. Pedometers register steps taken during walking and running activities and have been popularized as a motivational tool to encourage sedentary or inactive individuals to become more physically active. PA targets in “steps per day” including the commonly used adult benchmark of “10,000 steps,” is well-understood by the lay public. Recent interest in the use of pedometers includes the relevance of cut-points, in particular their equivalence with EE in different populations. The rationale for targets and cut-points, including 10,000 steps/day, has been challenged as a recommendation for some groups (77). For example, 30-min of brisk walking per day equates to 3–4000 steps or ~1250–1550 steps/km (78). Table 1, modified from Tudor-Locke and Bassett (4), uses data from a range of studies and can also be used to categorize PA level based on step counts.

**Table 1:** **Number of steps per day and corresponding physical activity level**.

Physical activity level	Steps per day
Sedentary or inactive lifestyle	<5000
Low active	5000–7499
Somewhat active	7500–9999
Active	10,000–12,500
Highly active	>12,500

A large and increasing number of pedometers are available. As devices vary widely in sophistication of function and reliability, readers are referred to a number of relevant publications for further information (79–83).

Objective evaluation of pedometers has identified numerous shortcomings in accuracy. For example, pedometer step counts are more inaccurate at slow speeds (<60 m/min) (84) therefore, may be inappropriate for work with older adults (85, 86). Pedometer readings can also vary according to where the pedometer is mounted or “worn” (84, 87, 88). For example, in centrally obese individuals with a large waist circumference, pedometers may rotate if worn in the standard position on the waistband (89, 90). Foot strike also varies between and within individuals so that when a pedometer is worn by different individuals, it may register a different step count for the same number of actual steps taken, presumably due to a differential between the foot strike of left and right legs (91).

Most pedometers fail to account for individual differences in height and leg length. Step count is also influenced by stride length (commonly related to both height and leg length) and speed of walking (87). If an individual walks faster than normal, a pedometer may underestimate total distance walked. Alternatively, pedometers may overestimate distance when walking slower than customary unless there are commensurate relative changes in stride length and step frequency when speed changes. The implications are that step count should not be used as a proxy for distance traveled without calibration of the pedometer to know how many steps an individual takes over a given distance. Calibration at a range of speeds may also be warranted. The ActiGraph accelerometer, the anterior mounted New Lifestyles, and the Walk4Life motion sensors have acceptable step count error values during treadmill walking at a range of speeds (84).

Pedometers have also been criticized for their ability to be manipulated to increase the total number of steps recorded. Simply shaking many devices will increase step count when the movement is clearly not associated with walking steps. Similarly, young people and other inquisitive participants may regularly look at the number of steps taken and alter their “typical” pattern of activity in order to increase the step count. To avoid or discourage such manipulation researchers often tape over the device to reduce any feedback based on step count. To increase the likelihood that monitoring is a reflection of a typical day, researchers often discount data collected on the first and last few days of a block of measurements.

Major advantages of pedometers are that they are relatively inexpensive, easy to use, and output data can be used to raise awareness regarding level of PA, including motivation for increased PA. As walking is such a common form of light- to moderate-intensity PA, a good measure of distance and speed is important. Similarly, if pedometers are used in an intervention or as a tool to monitor changes in daily PA, the sensitivity of the tool to measure change should be high. Tudor-Locke and Myers (92) reported that pedometers were able to track modest increases in walking volume in obese sedentary adults involved in an intervention, whereas PA diaries were not sensitive to the change in ambulation. Unfortunately, many sub-standard pedometers are available therefore selection of device for reliable measurement of steps should be considered carefully.

### Accelerometers

Accelerometers are motion sensors that detect accelerations of the body. Acceleration is defined as the rate of change in velocity over a given time; therefore, the frequency, intensity, and duration of PA can be assessed as a function of body movement (93). Accelerometers consist of piezoelectric transmitters that are stressed by acceleration forces. This leads to the production of an electrical signal that is subsequently converted by processing units to produce an indication of movement (15).

Accelerometers have gained considerable popularity in recent years as an objective approach to measure daily PA and represent a substantial improvement over self-report methodologies (3, 6, 94, 95). Accelerometry enables an estimation of intensity and duration of movement and the relationship between accelerometer counts and energy cost allows PA to be classified by intensity (13, 95–97). Numerous papers (5, 98–102) have reported that accelerometers are objective, practical, non-invasive, accurate, and reliable tools to quantify PA volume and intensity with minimal discomfort (95, 103–105). Technological advancements in accelerometry mean that the popularity and accessibility of this methodology has increased steadily such that it may be the preferred objective approach to assess PA and EE (95, 106, 107).

There is also increasing interest in the objective measurement of sedentary behaviors (or physical inactivity) (108). Sedentary behaviors include purposeful involvement in activities involving minimal movement and low levels of PA (108–110). However, this review does not detail the measurement of sedentary behavior and readers are referred to a number of recent publications in the area (111, 112).

Accelerometers provide information (outputs) regarding body movement in counts per unit time (referred to as an epoch). Importantly, movement counts have no biological meaning *per se* and must be converted to more relevant constructs (108), typically based on intensity. These include moderate-to-vigorous physical activity (MVPA) or sedentary behavior, or direct observation of PA, or some health outcome in calibration studies (113, 114). Rate or intensity of movement is captured using piezoelectric sensors to detect acceleration in one plane (uni-axial), two planes (bi-axial), or three orthogonal planes (tri-axial) representing vertical, anteroposterior, and mediolateral directions (15, 101). Accelerometers are more sophisticated and therefore superior motion sensors than pedometers.

Examples of uni-axial accelerometers include the ActiGraph wGT3X-BT (Actigraph LLC, Pensacola, FL) and Personal Activity Monitor – PAM AM200 (PAM B.V. Doorwerth, Netherlands) and bi-axial accelerometer – ActiTrac (IM Systems, Baltimore, MD). These sensors are typically worn so that the sensitive axis is oriented to measure vertical acceleration and deceleration (101). Accelerometers such as Actical (Philips Respironics, Bend, OR) can record movement in all directions however are most sensitive in the vertical plane (93, 101). In contrast, tri-axial accelerometers such as the Personal Activity Monitor – PAM AM300 (PAM B.V. Doorwerth, Netherlands) and RT6 (Stayhealthy Inc., Monrovia, CA) measure movement in three planes (15).

Tri-axial accelerometers arguably provide a more comprehensive assessment of movement representative of PA than uni-axial accelerometers. This may be particularly the case in children as the devices may be more sensitive to some activities including climbing and jumping (72). Hendelman et al. (85) reported that tri-axial accelerometers have a higher correlation than uni-axial devices with EE in adults.

Major advantages of accelerometers include their relatively small size and capacity to record data continuously over an extended period (days or weeks) (11, 15, 107) and lack of visual feedback to the individual wearing the device. The lack of immediate feedback means that the likelihood of an overestimation of PA (such as is possible with the manipulation of a pedometer) is reduced. Simple uni-axial accelerometers contrast with more sophisticated and precise tri-axial devices, particularly regarding estimation of EE (11).

Accelerometry has enabled both PA and sedentary behaviors to be measured with greater accuracy and precision (115, 116) than in older studies using subjective methodologies to quantify PA (3, 108, 117). A number of large-scale studies have successfully used accelerometry to assess PA in youth (116, 118) and increasingly accelerometry has been utilized in studies of very young children (104).

Accelerometry is based on the fact that speed is the change in position with respect to time, and acceleration is the change in speed with respect to time. Acceleration is typically measured in gravitational acceleration units (g; 1 g = 9.8 m/s^2^). When acceleration is zero, speed does not change, however, movement may still occur but at a constant speed. Because acceleration is also proportional to the net external force involved, it more directly reflects the energy costs associated with the movement. Accordingly, measurement of PA using acceleration is preferred to using speed. More detailed technical aspects of accelerometry are also not the focus of this review but are available from a number of authoritative sources (15, 119, 120–122).

Despite a good linear relationship between accelerometer counts and EE during walking, some concerns have been reported in studies of running. For example, Brage et al. (123) assessed the reliability and validity of the CSA (model 7164) accelerometer (MTI) across a wide range of walking and running speeds in laboratory and field settings and noted that the CSA output rose linearly (*R*^2^ = 0.92) with increasing speed until 9 kph but remained at ~10,000 counts/min during running. Therefore, oxygen uptake is underestimated at speeds >9 kph. Authors proposed that the lack of linearity may be due to a relatively constant vertical acceleration in running. Rowlands (101) reported similar findings from comparisons of uni-axial (ActiGraph) and tri-axial (RT3) devices and confirmed that activity was underestimated as speed increased using a uni-axial device with acceleration only assessed in the vertical plane. Rowlands (101) found that output from tri-axial devices was strongly related to speed, a reflection of the predominance of horizontal acceleration at higher speeds. Improvements in technology have resulted in single devices (124) measuring and reporting movement in all three axes separately and simultaneously (RT3). This study assessed whether using three planes of acceleration signals is superior to using only the vertical plane of the same unit for predicting AEE during locomotion and activities of daily living (ADL). The tri-axial capacity did not significantly improve the relationship between movement counts and AEE compared with uni-axial devices.

Another important comparison is between accelerometry outputs and indirect calorimetry. The RT3 motion sensor overestimated AEE for treadmill activity by 9% and underestimated ADL by 34%. The RT3 underestimated activity with greater upper-body movements by 24–64%. Compared to DLW assessed over 15 days and using the proprietary algorithms, Maddison et al. (125) found the RT3 underestimated AEE by 15% on average. While the RT3 provided a relatively accurate assessment of free-living AEE at the group level, it generally underestimated the AEE compared to DLW. These studies demonstrate that is not sufficient to only consider the number of axes used, but rather the technology inherent in the device or the data processing available. Plasqui et al. (126) noted that age, body mass, and height collectively explained 64%, while the tri-axial accelerometer (Tracmor) added only an additional 19% of the variation in TEE. In some studies, it is possible that most of the variance is explained by participant descriptors and the accelerometry data may have only marginal additional value. Few studies have provided data to demonstrate the ability of the accelerometer to predict individual AEE rather than AEE on a group level only; standard errors or limits of agreement should be presented. Plasqui and Westerterp (120) outline an important range of issues for consideration when comparing the validity of different accelerometers.

Irrespective of the use of accelerometry counts to convert to EE, a major advantage of the technique is the ability to quantify time spent in activities of different intensities. Accelerometer outputs are typically related to standard thresholds for activity of light, moderate, and vigorous-intensity (127–129). However, a major issue in the field is how to select cut-off points to define activity intensities. Despite a number of proposed cut-offs for some devices, there is currently no consensus (3, 130). If there is inconsistency in the use of accelerometers and cut-offs to delineate exercise or PA intensity, it is extremely difficult to make meaningful comparisons between the findings of different studies (106). Similarly, uniform cut-off points may not be truly representative of the same exercise intensity across individuals.

The most widely used accelerometer in studies of children and adolescents is the ActiGraph; however, the ranges for defining the lower limit for different intensities are considerable (3, 75, 131, 132). Most studies (133–135) have used a lower threshold for moderate PA and vigorous PA as suggested by Trost et al. (73), whereas some studies (136) have used a significantly higher cut-off point for moderate and vigorous-intensity PA as suggested by other validation studies (131, 137).

Accelerometers are most commonly worn on a waist belt and aligned with the right anterior axillary line and worn at all times for up to 7 days except when showering, bathing, swimming, or involved in contact sports. It is also useful to encourage participants to keep a record of when they removed the monitor for any reason. A common epoch length is 30-s with a minimum wear time defined as 10-h/day for 4 of the 7-days, one of which must be a weekend day (138). Non-wear time should also be specified, for example 60-min of consecutive zero counts. Total counts and minutes spent in sedentary, light, moderate, and vigorous-intensity PA can be calculated using the Actilife 5.5 software (ActiGraph, Pensacola, FL, USA) plus published cut-offs, for example, as described by Troiano et al. (139) for adults. In adults, the hip-mounted ActiGraph has demonstrated high inter-device reliability (*r* = 0.98) (123) and validity against indirect calorimetry (*r* = 0.56, *p* < 0.001) (129) and least variability when compared with other accelerometers (140).

Accelerometers have low sensitivity to sedentary activities and are unable to register static exercise (122). Similarly, as accelerometry is insensitive to PA that does not involve a transfer of the center of mass at a rate relative to the energy expended (e.g., weight lifting, walking up a slope, walking, and carrying a load), this will lead to errors in measurement of TEE (129, 141).

Like all methodologies, accelerometers have a number of limitations including the possibility of reactivity based on the individual knowing they are being monitored (3). If accelerometers are used for a limited number of days, these may not be representative of the individual’s habitual PA. Because accelerometers display inter- and intra-monitor variability, it is strongly recommended that trials are undertaken to identify outlying monitors and understand the inter-monitor variability before commencing a study (142). Further, as outlined above, accelerometers should be calibrated to each individual user. To improve the categorization of movement, some studies have used multiple accelerometers worn on different body parts (trunk, chest, wrists, legs, and feet). Whole-body EE can be determined from the composite of movements. Heil et al. (143) developed good guidelines and a very useful decision-making algorithm associated with accelerometry. Similarly, Mannini et al. (144) developed an algorithm to process wrist and ankle raw data from a single accelerometer to classify behavior into ambulation, cycling, sedentary, and other activities.

In very young children (<5 years), accelerometers and direct observation are well-established as the preferred PA monitoring approaches (96, 104) as they can detect short bursts of activity typical at this age (104, 145). However, the validity, reliability, and feasibility of accelerometry in toddlers (<3 years) have only recently been addressed (103, 145) and much more work is required in this area (107). The study by Van Cauwenberghe et al. (107) was one of the first to assess the feasibility of ActiGraph accelerometer assessment of PA in toddlers and the appropriateness of using accelerometer cut-points proposed for older children. ActiGraph accelerometers are the most widely used for PA research in children and adolescents (108, 145, 146).

In studies with young children, accelerometers have been programed to record data every 15-s (101, 103, 108) and Meterplus 4.2 software used to screen and clean data (Santech). It is also commonplace to omit data from both the first and the last day of the registration period (97) and periods of >10-min of consecutive zero activity counts regarded as non-wearing time and excluded (97, 103, 147).

The minimum number of minutes with recorded accelerometer data (registration time) required to constitute an eligible weekday and weekend day has been determined by defining the period during which at least 70% of the study population had recorded accelerometer data and 80% of that observed period constituted the minimum registration time (97, 102, 147). Days on which participants did not achieve the minimum registration time are considered as non-eligible days and are excluded. Minimum registration time has been defined separately for weekdays and weekend days as this often varies (101–103). If data are available for three valid days, this information is analyzed (103).

ActiGraph cut-points to define PA intensities for 3–5 year-olds (148, 149) are as follows: sedentary behavior: ≤37; light physical activity (LPA): 38–419; and MVPA: ≥420. Cut-points for 3-year old children (132): sedentary behavior: ≤301; LPA: 302–614; MVPA: ≥615. Cut-points by Van Cauwenberghe et al. (147) for 5-year old children were: sedentary behavior: ≤372; LPA: 373–584; and MVPA: ≥585.

Van Cauwenberghe et al. (95) found that the Pate cut-points can be used to classify sedentary and non-sedentary behavior in toddlers. However, none of the three sets of cut-points for preschool children appeared suitable to differentiate light and moderate-to-vigorous PA in toddlers. Without suitable equations for toddlers, Van Cauwenberghe et al. (95) suggest that accelerometer counts (i.e., counts per minute) be used as a measure of PA participation.

### Combined approaches

As no single technique is able to quantify all aspects of PA under free-living conditions, use of multiple complementary methods is recommended (13). For example, a potentially powerful approach to quantifying EE is the simultaneous use of accelerometry and HR monitoring (5, 11, 73, 141, 150). The rationale for combining these techniques is that accelerometer counts verify that elevations in HR are due to PA. As mentioned above, the relationship between HR and EE is influenced by a host of factors including age, gender, level of training, stroke volume, temperature, etc. (151). Limitations of accelerometry include the inability of the device to account for additional load carried by the user (5) and changes in the grade of the exercise surface. The fact that the two sets of limitations are completely different suggests that a more precise estimation of EE may be possible with a combined approach (141).

As mentioned in the section on HR monitoring, the HR–VO_2_ relationship is only linear during moderate to high-intensity exercise (152). Therefore, HR monitoring is useful to quantify PAEE but not the low-intensity PA characteristic of a large proportion of the total daily PA undertaken by many individuals. Accelerometry may be considered as the reverse, very limited in the assessment of AEE but able to quantify low levels of PA or sedentary behavior (73). As a combined HR and movement sensor, Actiheart exploits the advantages of each technology in a single device. Actiheart has been validated in adults during treadmill walking and running for the prediction of PAEE (153) and Corder et al. (5) has confirmed the same in children. Examples of combined movement counter and HR monitor include Actiwatch/Actiheart/Actiband; and ActiTrainer. A number of the more complex devices, including the SenseWear Armband, are discussed in a following section.

Examples of prominent international companies producing HR monitors include Polar^1^, Suunto^2^, and Garmin^3^. A growing range of products with more sophisticated features are available and not discussed here. These include the capability of collecting data on both physiological and mechanical variables including distance, speed, altitude, cycling power output, running pace and cadence, altimeter features, GPS features, and cycling and running features. Increasingly, smartphone applications will provide the new opportunities for monitoring, including the quantification of non-wear time of objective devices such as accelerometers (154, 155). Discussion of these new approaches is beyond the scope of the current review.

### SenseWear armbands

The SenseWear armband^4^ collects data from multiple sensors: a skin temperature sensor, near-body temperature sensor, heat flux sensor, galvanic skin response sensor, and a bi-axial accelerometer. The combination of signals from these sensors enables the assessment of activity type and intensity and the incorporation of detail regarding age, gender, height, and weight enables EE to be estimated using proprietary algorithms. Readers are referred to a number of recent validation studies (156–158).

### Intelligent device for energy expenditure and activity

The intelligent device for energy expenditure and activity (IDEEA) (MiniSun) estimates EE from 35 postures and activities and it can identify and record (159) using multiple sensors. A detailed overview of the device is available in the papers by Zhang et al. (160, 161). Much of the published work using the device has been laboratory-based, either validation studies of EE (160) or highly controlled, short duration gait and posture analyses (161–163). The device has also been used to measure patterns of PA and estimate EE over 3 days in a free-living subset of participants in the DiOGenes study (164). A comprehensive assessment by Whybrow et al. (159) evaluated the IDEEA to estimate EE against both whole-body indirect calorimetry, and DLW. The study also compared EE estimates from measured and estimated calibration values.

Compared to indirect calorimetry reference methods, Whybrow et al. (159) reported that the IDEEA significantly overestimated EE outside the laboratory but this overestimate is not always apparent under controlled conditions (161). Using the IDEEA in a calorimeter, Whybrow et al. (159) found the device underestimated the EE of slow walking and overestimated faster walking. In contrast, others have reported better agreement when used in a more natural walking motion (165), including a treadmill, than was possible in the calorimeter.

Limitations of the IDEEA system include the relative difficulty of attaching the sensors, the inconvenience and discomfort of wearing the sensors, and the limited memory capacity. Further, the device is unable to detect cycling as an activity and usually allocates a stationary sitting or standing posture with an estimated EE unrelated to the work completed. Consequently, the IDEEA underestimates the EE of cycling (159) and overall is of similar accuracy in estimating EE to the HR method.

To avoid the cost associated with using multiple devices, and participant and researcher burden, a number of studies have paid greater attention to the identification of activity types based on acceleration data measured with a single accelerometer (166, 167). Bonomi et al. (168, 169) measured ADL with a single accelerometer in a population of healthy adults and used a decision tree algorithm to identify the activity types performed. The decision tree evaluated attributes (or features) of the acceleration signal. Using DLW as the criterion measure, the identification of types of PA such as lying, sitting or standing, active standing, walking, running, and cycling combined with a simple methodology to define the intensity of activity type, improved the estimation of TEE, AEE, and PAL compared with activity counts.

In summary, improvements in individual and/or multiple sensors has progressively improved the objective assessment of physical activity and energy expenditure. For example, Lyden et al. (170) very recently reported a method to improve the estimation of both free-living active and sedentary behaviours from an accelerometer and validated against direct observation. One of the better studies to assess energy expenditure estimation of several activity monitors consistent with a 4-h stay in a room calorimeter was undertaken by Dannecker et al. (171). IDEEA and Directlife estimates of energy expenditure were not different to measured energy expenditure. In this study, Actigraph and Fitbit devices significantly underestimated energy expenditure. Adam Noah and colleagues (172) assessed the new Fitbit accelerometers against an indirect calorimetry system (Cosmed K4) and the commercially available Actical devices. They also found that Fitbit underestimated energy expenditure in a number of activities however suggested the device was reliable and valid for monitoring over-ground energy expenditure.

## Selected Subjective Measures

A range of subjective approaches complement the objective measures discussed above. Subjective methods are indirect approaches and typically involve the individual recording his or her own activity (93). These include direct observation, activity diaries, PAQs, and interviews (66), however, here we only address some examples of such approaches. Each approach assesses different dimensions of PA, has a variety of outcome variables, and associated strengths and weaknesses.

The fundamental shortcoming of all subjective approaches is the potential to influence the voracity of the information collected (173). Questionnaires rely on one’s recollection of PA and enable a categorization of individuals from sedentary and inactive, through to active or very active. Simply stated, subjective approaches quantify the perception of PA as opposed to PA *per se* (108) as typically questionnaires lack the precision needed to detect PA changes on a day-to-day basis (174, 175).

Because of the absence of inexpensive, readily available, non-invasive, valid, and reliable objective technology for the assessment of AEE in large numbers of free-living individuals, many researchers rely on estimates of AEE derived from PAQs (176). However, most PAQs have demonstrated limited reliability and validity (173) and it is questionable whether any are valid for estimating AEE at the individual and group levels (176).

Valid measurement of PA is also very challenging in some populations, including children <5 years of age, mainly due to the sporadic and intermittent nature of their movement (100, 104, 108). Proxy reports from parents can be useful for rank ordering young children on PA behavior (97), however, inaccurate estimates of the amount and intensity of PA is a primary concern with this approach (103, 104). Cognitive limitations of children younger than 10 years of age also limits the accuracy of questionnaire-derived PA data from young children (114), therefore proxy reports are commonly used (100).

At the individual level, the validity of data derived from subjective approaches for the calculation of PAEE and MVPA is questionable compared to objective alternatives such as accelerometry and the DLW technique that enable data collection over an extended period of time (106, 177). The choice between subjective and objective approaches is typically based on the resources available and the number of participants required for the study. Subjective data from PAQs tend to be more valid at a group level and this combined with the relatively low cost, means that a larger number of people can be surveyed or assessed (11, 14).

A fundamental problem in the field is when data derived from a self-report PAQ are converted to units of EE. An appreciation of the variability in MET value is important here. Clearly, it is not feasible to measure the energy cost of activities for each individual in large epidemiological studies (176), hence the Compendium of physical activities is widely used (17, 58, 59). An important limitation of the Compendium is the reliance on group averages based on assumed body weight or REE and therefore energy equivalents that may not apply to individuals (18, 178, 179).

In youth, there is no standardized reference to parallel the 1 MET equivalent of 3.5 mL/O_2_/min for adults (106) despite possible differences for MET multiples for the same activities in children and adults (180). Estimations of EE from self-report PAQs in youth typically use adult-derived standard energy costs of specific activities (17). However, these can be adjusted for the higher REE in children (181). Ridley et al. (182) have developed a Compendium of EE for youth, however, only 35% of the values in this Compendium are derived from data measured in young people with other values extrapolated from adult data (183). Recent published literature has also provided guidance regarding the relative merits of different PA assessment tools for use in defined populations, including overweight and obese children (183).

Self-report PAQs are often the only feasible way to assess PA in many situations (106, 176) particularly in studies involving a large number of participants. Similarly, self-report approaches are important for the assessment of aspects of PA not easily measured objectively, including mode and domain (184). Some PAQs can accurately determine the mode of activity and can be used to rank, group, or categorize PA levels with a degree of confidence (185, 186).

While some studies contend that it is possible for self-report PAQs to assess some aspects of MVPA (187, 188), most agree that PAQs are less accurate than objective methods for estimating PAEE and MVPA (11, 106, 189).

Relatively, few validation studies of PAQs have used DLW (185, 186) with accelerometry being the most common criterion (184, 188, 190). Corder et al. (106) were the first to simultaneously assess the validity of estimated PAEE and MVPA from PAQs against both DLW and accelerometry. This approach enabled the assessment of the strengths and weaknesses of self-reported PA not possible when only one criterion method is used. A major finding was that all PAQs consistently underestimated PAEE (106, 177). Readers are also referred to a paper by Patterson (191) for an explanation of associations between questionnaires and objective measures and a multi-step process of developing an instrument to measure a construct such as PA. Too often researchers refer to validation when a questionnaire is correlated with an objective measure, however, this does not represent criterion-related evidence of validity (177).

A comprehensive appraisal of the use of PAQs to assess AEE in population-based studies by Neilson et al. (192) is particularly informative. They concluded that despite numerous validation studies (using DLW), the validity of PAQs for AEE estimation remains unclear. Neilson et al. (192) contend that weaknesses in study design and reporting of studies is a major contributing factor, along with a failure to make PAQs freely available. Some reasons for discrepancies in estimates of AEE between PAQs and DLW could include the failure of the PAQ to incorporate key activities and differences in length of time assessed in each approach (192).

### Physical activity records or diaries

Records or diaries can provide very detailed information regarding activity types and patterns, for example walking, watching television, etc., purpose: for example PA, exercise, occupational, transport, etc., intensity (light, moderate, vigorous), duration (in minutes or hours), frequency (how often), and mode or body position (for example sitting, standing, walking) (2). Detailed recordings enable one to account for PA (or EE) undertaken in relatively short 15-min time intervals. Over a 24-h timeframe, one can determine how long an individual engaged in various activities and subsequently translate this into EE predictions using MET values for each task and intensity level (2). As mentioned above, a wide range of physical activities have been coded and published in a Compendium of physical activities that categorizes each task or activity into domains and intensities (commonly based on METs). The Compendium was developed in 1989 and extensively updated in 2000 and again in 2011. A disadvantage of activity diaries is the high participant burden, including the time needed for individuals to record physical activities in blocks of 15 min across multiple 24-h periods.

### Multimedia activity recall

A well-developed use of time measure is the Multimedia Activity Recall for Children and Adults (MARCA). The MARCA is a computerized 24-h recall tool that asks participants to recall their use of time in the previous day from midnight to midnight using meal times as anchor points across the 24-h period day (138). Participants recall their day in blocks of 5-min or more by choosing from 520 activities organized under different categories including “Self-Care,” “Occupation,” and “Sport/Recreation.” Each activity in the MARCA is assigned a MET value to estimate EE based on an expanded version of the Ainsworth Compendium (17, 58).

The MARCA was originally designed for use in children and adolescents (193) but has been modified for use in adults (194). The adult version has test–retest reliabilities of 0.990–0.997 (*p* ≤ 0.0001) for MVPA, PAL (average daily rate of EE in METs), sleep and screen time, and convergent validity between PAL (estimated average rate of EE) and accelerometer counts/minute of ρ = 0.72 (186). A recent comparison with the DLW technique displayed correlations of ρ = 0.70 for TEE (195).

The MARCA enables a determination of EE based on minutes spent in the following zones: 0.0–0.9 MET (sleep); 1.0–1.9 METs (very light physical activity, VLPA); 2.0–2.9 METs (LPA); 3.0–5.9 METs (moderate physical activity, MPA); and ≥6 METs (vigorous physical activity, VPA). A time-use profile is determined by minutes spent in major “activity sets” including physical activity, computer, active transport, passive transport, quiet time, self-care, socio-cultural, work/study, chores, sleep, and TV/videogames.

### Physical activity questionnaires

Physical activity questionnaires are the most widely used approach to monitor activity levels (14). Global questionnaires are easy to administer and complete; however, most only provide minimal information about activity and simply enable a group to be categorized as “active” or “inactive.” Recall questionnaires are longer and provide more detailed accounts of PA including information about frequency and duration of activities over extended periods. The significant variability in PAQs includes the amount of detail provided, length of time assessed, and the extent of supervision required to successfully complete the questionnaire (173). Neilson et al.’s (192) systematic review of PAQs provides a comprehensive appraisal of the strengths and weakness of PAQs and their ability to quantify AEE in relation to DLW.

Physical activity questionnaires are completed over a minimum of a 24-h period and up to 7-days (184). PAQs are commonly more valid when administered in an interview context, either by telephone or face-to-face, however, are notorious for overestimating vigorous PA and underestimating time spent undertaking ADL (2). Miscalculation of the total volume or intensity of PA may have different implications, for example, in the determination of the dose–response relationship between PA and health. Therefore, it is highly desirable to include objective measures with self-report instruments to minimize intentional or unintentional misreporting of PA. Welk et al. (196) recently reported on the validity of a 24-h PA recall compared with EE using SenseWear Armbands. There was good agreement between approaches; however, PA recall may result in biased estimates of MVPA in adults at the group and individual level. If a combined approach is not possible in the whole cohort, it is highly recommended that both measurement approaches be used in a representative sub-sample. Strengths and limitations of PAQs, with specific reference to individual questionnaires, are outlined in Table 2.

**Table 2:** **Strengths and weaknesses of self-report PAQs**.

Strengths	Limitations
∙ Able to measure large numbers of participants at low cost ∙ Theoretically, recall does not alter behavior ∙ A variety of dimensions of PA can be assessed ∙ Can extrapolate to EE ∙ Suitable for a wide range of populations ∙ Measurement tool can be adapted to suit the population ∙ Possible to compare results from different locations when using the same instrument, e.g., IPAQ	∙ Recall challenges for some populations, e.g., children and the aged ∙ Semantics may be an issue, e.g., terms like “moderate-intensity” may be ambiguous ∙ Dependent upon response rates and ability of participants to follow instructions ∙ Completeness of answers ∙ Activity choices listed in questionnaire may not be relevant for some populations ∙ Minimum amount of detectable change (sensitivity)

## Conclusion

With so many approaches available, the accurate assessment of PA and quantification of EE can be very challenging. It is important to appreciate that irrespective of the apparent sophistication of techniques, all have inherent strengths and weaknesses. A better understanding of the merits of different approaches should inform decision-making and selection of the best approach for the situation, including for specific populations such as overweight and obese children (183).

Objective measurement approaches have been the primary focus of this review; however, subjective approaches, including diaries and PAQs, have the potential to provide rich descriptive data. However, an acknowledged major shortcoming of subjective approaches is their heavy reliance on an individual’s recollection of events. Therefore, depending on the context, such approaches may be prone to under- or over-reporting.

Despite the advantages of objective measures, some approaches and devices, including accelerometry, are inappropriate for the quantification of activities other than walking and running. For example, accelerometers are not able to quantify movement in swimming and cycling.

The ideal scenario is for PA and EE to be measured with research quality tools and approaches. If one is interested in total PA, an understanding of physical inactivity is also important. The measurement of total PA or EE is informative but much richer information is available when PA and exercise intensity is monitored, not simply the total dose of PA undertaken. One of the fundamental shortcomings of any approach, again irrespective of the level of sophistication, is the integrity of the device. This includes the reliability of each instrument and whether the device has been calibrated to each individual for maximum benefit.

In summary, PA is a complex construct encompassing different dimensions, such as PAEE or MVPA; a range of contexts such as occupation, transportation, exercise, and daily activities; and different types of activity or exercise (177). Given the variety of applications for measures of PA, for example, in surveillance, epidemiology, clinical, and intervention research, it is highly unlikely that a single measure of reported PA would suffice, indeed be achievable. As mentioned in an earlier section, significant advances have been made with approaches that combine objective measures such as accelerometers, HR monitors, and geographic location sensors with self-report of context and purpose, sometimes reported in real time (154, 177).

Troiano (177) has provided sound advice regarding improvements to self-report approaches, including the suggestion that consideration of study or project requirement influence the choice of assessment instrument. Specifically, what aspect(s) of PA does one wish to measure, what are the characteristics of the target population, and will the data be used to describe groups or individuals? Secondly, Troiano (177) suggests that findings from self-report instruments should be addressed as “reported PA” in recognition that what has been reported may not “precisely and accurately reflect the behavior being sought.”

## Author Contributions

All authors made substantial contributions to the conception and design of the paper. Andrew P. Hills drafted the review and Najat Mokhtar and Nuala M. Byrne revised it critically for content. All authors approved the final submission of the document and agree to be accountable for all aspects of the work.

## Conflict of Interest Statement

The authors declare that the research was conducted in the absence of any commercial or financial relationships that could be construed as a potential conflict of interest.

## Footnotes

^1^http://www.polar.fi

^2^http://www.suunto.com

^3^http://www.garmin.com

^4^http://www.bodymedia.com
